# A Foot in the Door for Dermatophyte Research

**DOI:** 10.1371/journal.ppat.1002564

**Published:** 2012-03-29

**Authors:** Rebecca Rashid Achterman, Theodore C. White

**Affiliations:** 1 Department of Biology, Western Washington University, Bellingham, Washington, United States of America; 2 Division of Cell Biology and Biochemistry, University of Missouri at Kansas City, Kansas City, Missouri, United States of America; Duke University Medical Center, United States of America

## What Diseases Do Dermatophytes Cause?

**Figure 1:**
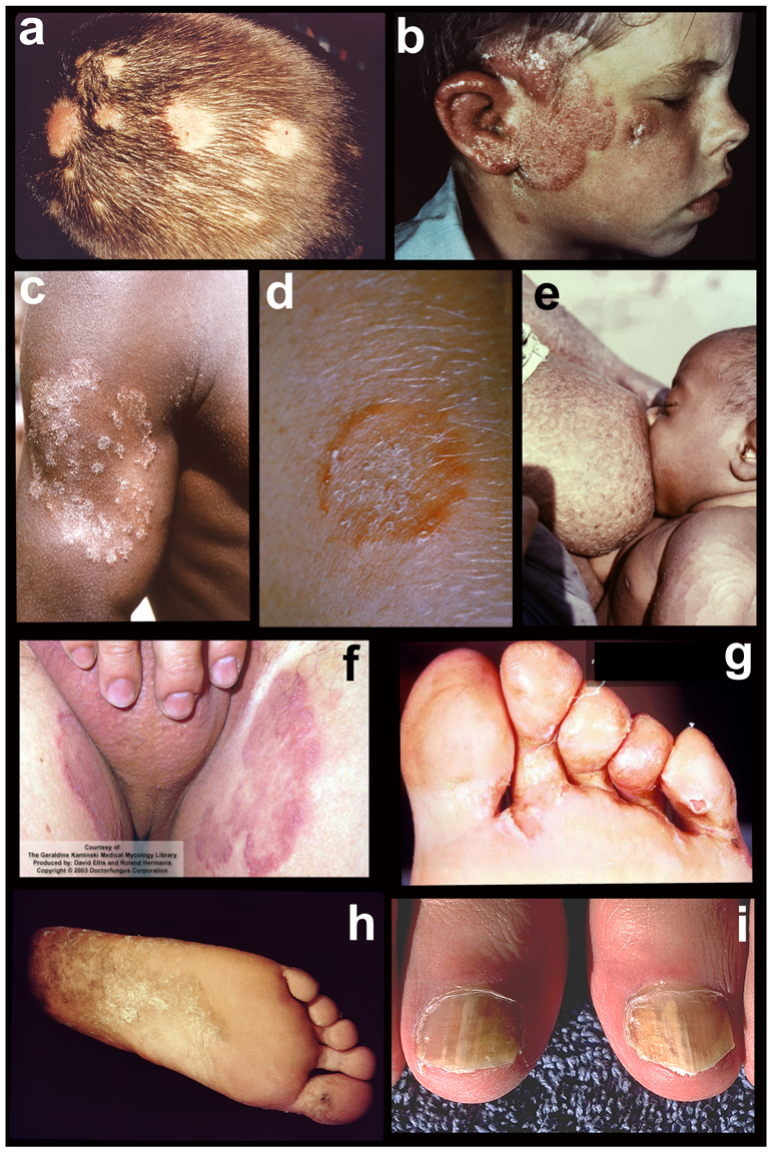
Tineas throughout the body. Tineas are dermatophyte infections of the skin. From top to bottom, left to right: fungal infections of a) hair (tinea capitis); b) face (tinea capitis / ringworm); c) arm (tinea corporis); d) close-up of ringworm; e) torso with concentric rings (tinea imbricata / tinea corporis); f) groin (tinea cruris); g) toe webbing (tinea pedis); h) foot (tinea pedis / “moccasin” type); and i) nails (onycomycosis). Photos courtesy of Doctor Fungus (http://www.doctorfungus.org) and the Public Health Image Library (PHIL, http://phil.cdc.gov/phil/home.asp).

**Figure 2:**
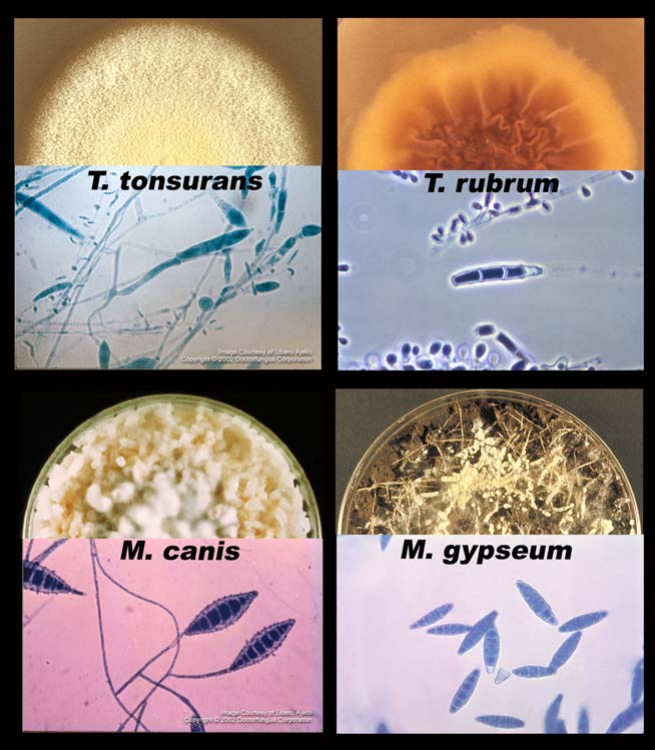
Major dermatophyte species—appearance in the laboratory. Each section shows one of four species of dermatophyte, including a semicircle of fungus growing on an agar plate (top) and a microscopic picture of the asexual spores (macroconidia and microconidia, bottom). Photos courtesy of Doctor Fungus (http://www.doctorfungus.org) and the Public Health Image Library (PHIL, http://phil.cdc.gov/phil/home.asp).

Dermatophytes are a group of filamentous fungi that cause infections of the skin (see Figure 1 for diseases, Figure 2 for typical dermatophyte species). Diseases caused by dermatophytes include athlete's foot, ringworm, jock itch, and nail infections (onychomycosis). The medical terminology for dermatophyte infections is to use the word tinea (to denote a fungal infection of the skin) followed by a word that describes the site of infection. For example, tinea pedis refers to athlete's foot and tinea capitis is scalp ringworm. In general, dermatophytes remain localized to keratinized surfaces such as skin, hair, and nails and do not invade deeper tissues. That said, dermatophyte infections in immunocompromised patients can be quite severe.

Dermatophytes are grouped into three general categories based on their natural environment: anthropophilic (live exclusively on humans), zoophilic (live on an animal host), and geophilic (live in the soil) [1]. The majority of human infections are caused by anthropophilic species; however, species from all three groups have been associated with human disease. For example, pets with ringworm can transmit the infection to their owners, and stray cats carrying dermatophytes are considered to be a vector for infection in several eastern and southern European countries [2].

## Is the Most Prevalent Disease the Same from Country to Country?

Although dermatophytes are found throughout the world, the most prevalent strains and the most common sites of infection vary by region [2], [3]. Hot, humid climates and overcrowding predispose populations to skin diseases, including tinea infections [4]. Developing countries have high rates of tinea capitis, while developed countries have high rates of tinea pedis and onychomycosis [2].

Low socioeconomic conditions are strongly linked to higher prevalence rates for skin infections, including tinea infections. A review of 18 studies representing large geographical areas determined that tinea capitis is present in up to 19.7% of the general population in developing countries [4]. A recent study found tinea capitis present in more than 30% of children at certain grade levels in some urban areas of the United States [5].

High prevalence rates of tinea pedis and onychomycosis have been linked to increased urbanization, community showers, sports, and the use of occlusive footwear [2], [3], [6]. These factors are thought to contribute to the high prevalence of tinea pedis in certain occupational groups, including marathon runners (22%–31% prevalence), miners (21%–72.9% prevalence), and soldiers (16.4%–58% prevalence) [2]. Several of these studies also found high rates of onychomycosis presenting with tinea pedis. Although tropical and subtropical climates have a higher overall prevalence of skin mycoses, tinea pedis and onychomycosis are rare in India and rural Africa [6].

## Why Can People Get Athlete's Foot (and Other Dermatophyte Diseases) More Than Once?

**Figure 3:**
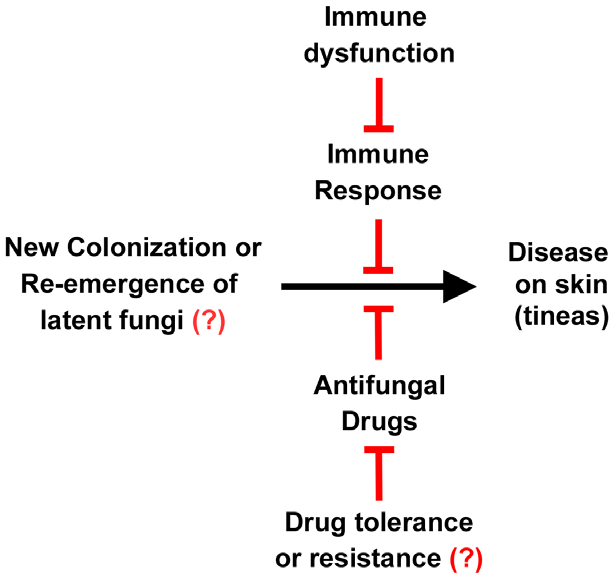
Relationship between fungus, disease, the immune system, and drugs. The left signifies the presence of dermatophytes on the skin, either by new infection or relapse of a previous infection. The right signifies tineas caused by these infections. This progression to disease can be inhibited by the immune system and antifungal drugs. However, immune dysfunction can reduce the immune response to these infections, and drug resistance or tolerance can overcome the action of the drugs. Resistance or tolerance to antifungal drugs is implied but is not documented in dermatophytes. The roles of the immune system, immune dysfunction, drug response, and drug resistance are all areas of active research.

Dermatophyte diseases recur at a high rate following treatment with an antifungal [7]. It is currently unknown whether this is due to insufficient clearing of the fungus during treatment and reemergence of disease, and thus an example of relapse, or if these represent new infections (Figure 3). The high false-negative culture rate from clinical samples contributes to this problem. The advent of molecular biology tools may provide a means by which clinicians can more accurately determine the presence (or absence) of dermatophytes [7]. Certainly, such tools will help determine whether a new infection is indeed caused by the same strain as a previous infection in the same patient.

Treatment of dermatophyte infections represents a significant cost burden. It has been estimated that over US$500 million per year is spent worldwide on drugs to treat dermatophytoses [8]. Treatment for skin infections is generally by a topical antifungal. If hair roots or nail beds are infected, an oral antifungal agent is generally prescribed. Nail infections are often recalcitrant to treatment. Immunocompromised patients can experience disseminated dermatophyte disease, which has a particularly high treatment failure rate (30.8%) [9].

## Why Aren't There More Drug-Resistant Dermatophytes?

Over-the-counter antifungals are commonly used to self-treat athlete's foot and jock itch. This might be predicted to lead to drug resistance. Surprisingly, drug resistance among dermatophytes is rare. Two large clinical studies looking at drug susceptibility in dermatophytes did not find significant increases in the minimum inhibitory concentration of several antifungal drugs used to treat dermatophytes [10], [11]. That said, occasional drug resistance has been documented. For example, a single amino acid substitution in the target enzyme was found to confer resistance to terbinafine in a clinical isolate from a patient with onychomycosis [12], [13].

The question remains, why aren't mutations conferring drug resistance a more widespread occurrence? One possibility is that dermatophytes are able to tolerate drugs without acquiring point mutations in the target enzyme. In the yeast *Candida albicans*, several mechanisms of resistance have been documented that do not require a point mutation in the target enzyme. More research is needed to determine if something similar is happening with dermatophytes.

## Why Don't We Know More about How Dermatophytes Cause Disease?

Despite the prevalence of dermatophyte infections worldwide, a sophisticated understanding of how they cause disease is lacking [14]. The historic difficulties in working with dermatophytes have been two-fold: technical difficulties due to poor virulence models and a lack of genetic tools, and an under-appreciation of the need to study these organisms.

There have been several recent advances that minimize the technical difficulties of working with these organisms. Performing genetics in dermatophytes has historically posed a challenge; however, recent advances in the field have provided a foundation for genetic manipulation of several species of dermatophytes [15], [16].

Currently, the most common animal model for studying dermatophyte virulence is the guinea pig. Although this has been useful for zoophiles, guinea pigs and other dermatophyte animal models do not provide accurate infection models for most anthropophilic species [15]. Other virulence models include determining the ability of the organism to grow on keratinized surfaces such as sterilized nail fragments, which is a non-quantitative model. Recently, skin explants have been used to study dermatophyte adherence and invasion. Human epidermal tissues are commercially available and represent a possible virulence model to study the initial stages of dermatophyte infection [15].

One other reason that we don't know more about dermatophyte disease is that most scientists, including many mycologists, do not consider dermatophytes as important as other infectious diseases. Therefore, there are a limited number of researchers working on the problem and there are limited funding opportunities. However, dermatophyte infections are likely to infect every person at least once in their lifetime [8]. While mortality due to dermatophytes is very low, there is significant morbidity associated with these infections, particularly in the armed forces and active adults. These infections are likely the most common fungal infection worldwide, with high rates of incidence and prevalence in most countries. In addition, the worldwide cost of dermatophyte treatment each year is over US$500 million [8].

## What's Next for Dermatophyte Research?

Dermatophyte research is poised to take off. The sequencing of seven dermatophyte genomes was recently completed, and the sequence information is now publically available [17], [18]. Analysis of the genome sequences demonstrates that a group of proteinases necessary for degradation of keratin is increased in number in the dermatophytes compared to closely related fungal species. These genome sequences, combined with better genetic tools and a promising model in which to study virulence, provide an optimistic outlook. Sequence information can be used to make informed hypotheses about which gene products, such as the proteinases, are important to virulence, and these genes can be deleted and tested in virulence models. These experiments will contribute to our understanding of how dermatophytes interact with human cells and cause disease. Knowing the fungal factors involved will allow development of better therapeutics and will inform preventative treatments.
